# Smartphone-Based Passive Sensing for Behavioral and Physical Monitoring in Free-Life Conditions: Technical Usability Study

**DOI:** 10.2196/15417

**Published:** 2021-05-11

**Authors:** Simone Tonti, Brunella Marzolini, Maria Bulgheroni

**Affiliations:** 1 Ab.Acus srl Milano Italy

**Keywords:** telemonitoring, data integrity, technical validation, cloud computing, ubiquitous computing, behavioral analysis, mHealth

## Abstract

**Background:**

Smartphone use is widely spreading in society. Their embedded functions and sensors may play an important role in therapy monitoring and planning. However, the use of smartphones for intrapersonal behavioral and physical monitoring is not yet fully supported by adequate studies addressing technical reliability and acceptance.

**Objective:**

The objective of this paper is to identify and discuss technical issues that may impact on the wide use of smartphones as clinical monitoring tools. The focus is on the quality of the data and transparency of the acquisition process.

**Methods:**

QuantifyMyPerson is a platform for continuous monitoring of smartphone use and embedded sensors data. The platform consists of an app for data acquisition, a backend cloud server for data storage and processing, and a web-based dashboard for data management and visualization. The data processing aims to extract meaningful features for the description of daily life such as phone status, calls, app use, GPS, and accelerometer data. A total of health subjects installed the app on their smartphones, running it for 7 months. The acquired data were analyzed to assess impact on smartphone performance (ie, battery consumption and anomalies in functioning) and data integrity. Relevance of the selected features in describing changes in daily life was assessed through the computation of a k-nearest neighbors global anomaly score to detect days that differ from others.

**Results:**

The effectiveness of smartphone-based monitoring depends on the acceptability and interoperability of the system as user retention and data integrity are key aspects. Acceptability was confirmed by the full transparency of the app and the absence of any conflicts with daily smartphone use. The only perceived issue was the battery consumption even though the trend of battery drain with and without the app running was comparable. Regarding interoperability, the app was successfully installed and run on several Android brands. The study shows that some smartphone manufacturers implement power-saving policies not allowing continuous sensor data acquisition and impacting integrity. Data integrity was 96% on smartphones whose power-saving policies do not impact the embedded sensor management and 84% overall.

**Conclusions:**

The main technological barriers to continuous behavioral and physical monitoring (ie, battery consumption and power-saving policies of manufacturers) may be overcome. Battery consumption increase is mainly due to GPS triangulation and may be limited, while data missing because of power-saving policies are related only to periods of nonuse of the phone since the embedded sensors are reactivated by any smartphone event. Overall, smartphone-based passive sensing is fully feasible and scalable despite the Android market fragmentation.

## Introduction

### Background

In 2020, smartphone users are approximately 3.5 billion people (ie, about the 45% of the world population). Smartphones are a widely spread resource that health care providers might extensively use to improve the quality and timeliness of service to the citizen at acceptable costs.

Potentialities of smartphones in health care are being widely explored [1]. A PubMed search of “smartphone” or “mobile phone” and “monitoring” for articles published between January 1, 2000, and September 30, 2020, found 5246 articles with 74.04% (3884/5246) published after January 1, 2015, demonstrating a continuous increase of interest in the last few years.

Furthermore, the growing number of available apps in the Google and Apple stores covers an increasingly large spectrum of services able to support most citizens’ daily activities. However, the effective diffusion of smartphone in the clinical practice is slowed down by social, organizational, and technical barriers [2]. Clinical practice requires the capability of continuously following up individuals along their care paths (longitudinal monitoring) assessing variations in time due to disease progression or intervention results. For this purpose, an underlying monitoring app must be robust and reliable and able to run on a wide base of smartphones in a totally unobtrusive and transparent way [3,4]. This approach addresses the ubiquitous computing paradigm that, through technologically transparent tools, enables the integration of small connected and inexpensive devices in the daily life of people. Transparency and density of the technological framework lead to higher levels of acceptability and reliability thanks to the reduced intrusiveness and, at the same time, the improved capillarity of the technology.

Meanwhile, the collected data must adhere to robust and device-independent quality standards to ensure measurement repeatability to generate clear clinical outcomes [5], while smartphone vendor policies contribute to increasing fragmentation due to strategic choices. Continuous monitoring apps and in particular passive sensing smartphone-based platforms must cope with constraints and limitations related to manufacturer choices and policies that need to be carefully assessed and cleared before large-scale deployment in health care with prevention and follow-up objectives.

On the other side, the level of engagement of the end user needs to be improved. Today, the longer the follow-up period, the higher the chances are for dropout [6]. Attrition rates from 30% to 70% are often reported [7-9]. Technological issues can dramatically impact the use in a daily routine.

Reliability and robustness are the most important drivers to ensure proper diffusion within the clinical practice; however, studies characterizing the smartphone-based platforms from this point of view are lacking. Many studies address the clinical relevance of the acquired data (see Prior Work section), but very few analyze the impact of technical issues on the scalability of the solutions in the daily routine and their performance in a heterogenous technical environment where hardware characteristics and proprietary policies have a strong impact on the quality of delivered data and calculated indexes.

### Prior Work

Mental health–related studies have widely investigated the use of smartphone-based sensing platforms to cope with the need of unobtrusive and continuous data collection while reducing biases in patient behavior. Dogan et al [10] provide a comprehensive review of the current status of the technological impact on affective disorder management. Several studies about the correlation between affective disorders and smartphone use are investigated, and technical problems, in particular issues related to different operating systems, are reported as the most common reasons for discontinuation. The use of smartphone-embedded sensors for health monitoring systems is analyzed by Majumder et al [11] who identify, as a main driver for successful penetration of these technologies, the availability of affordable apps compatible with the main mobile operating systems and devices from different manufacturers. Similarly, the need for apps with reduced battery drain and standardized performances regardless of the device brand is reported by Baig et al [12] and Yu et al [13], while Boonstra et al [14] define performance, interoperability, and battery consumption as the most impacting issues. Differing operating systems are reported as the leading cause of data loss. The data collection rate is still only 55% of the scheduled acquisition time for Android smartphones, indicating the need for additional development work to provide more stable and reliable tools. Finally, Hossain and Poellabauer [15] present the challenges encountered in building the CIMON (Crew Interactive MObile CompanioN) system, a continuous smartphone sensing app. This system is specifically designed for the iOS system, and the main issues reported are energy consumption, storage, and operational continuity. Nevertheless, because of Apple’s strict policy development limitations and terms, the variability in terms of technical policies between iOS devices is not even comparable with the Android market, which is required to deal with a broader pool of brands and proprietary management policies.

The need for robust and reliable passive sensing systems that exploit the smartphone as data collector is gaining relevance in the clinical debate, and recent studies [16] show a good correlation between behavioral data collected through smartphones and mental health–related scores [17-19] and also show how features calculated from smartphone data may capture a wider set of behavior descriptors not assessed by standard scores [20]. Other studies report strong correlations between smartphone-related nonmedical parameters, changes in lifestyle, and variations in mood [21]. In particular, frequency [22] and duration [23] of calls have been correlated to the onset of depressive symptoms.

### Goal of This Study

In this paper, we identify the main issues a smartphone-based monitoring app must resolve to be a suitable tool for longitudinal measurement of personal behavior on a diverse and continuously changing technological panorama.

A testing platform, QuantifyMyPerson (QMP), has been used for this study as it is a proprietary smartphone-based app that allows direct access to the collected raw data. QMP uses the embedded sensors of the smartphone itself and smartphone use information to provide 24/7 monitoring of the user’s life in terms of both physical and cognitive activities. The system architecture allows remote storage and processing of acquired data to be made available to the operator through a web-based dashboard. By design, QMP does not provide any feedback to the user and does not introduce any burden other than carrying the phone to avoid influencing the user’s behavior while unobtrusively capturing their life habits.

The aim of this study is to pinpoint the main technological issues encountered within an operating context and identify the most relevant aspects to be considered when a monitoring platform is deployed. The findings of this study will inform technical choices to reach scalable, usable, and reliable solutions that can reach large pools of users.

Despite the fact that the acquisition of data through passive sensing systems happens in the most transparent way, the collected information belongs to the user’s private sphere and there are privacy issues. Privacy and ethical issues are relevant perceived barriers in the spread of mobile health (mHealth) solutions and smartphone-based data collectors [24,25]. According to a recent review [26], broad consent and pseudonymization are frequently used approaches to manage these kinds of issues. A robust ethical framework is not yet clearly defined, and future evolutions should consider technical development, clinical benefits, and ethical issues together to shape an effective implementation of passive sensing in health care. Technical findings and outcomes of this study aim also to contribute to the definition of this framework promoting the use of passive data in an ethically safe and sound fashion.

## Methods

### Data Acquisition System

QMP is a composite system managing background acquisition of 24/7 data related to the social use of the smartphone (through call logs, app use, and device use) and to the user’s activity habits (through GPS and accelerometer analysis).

**Figure 1:**
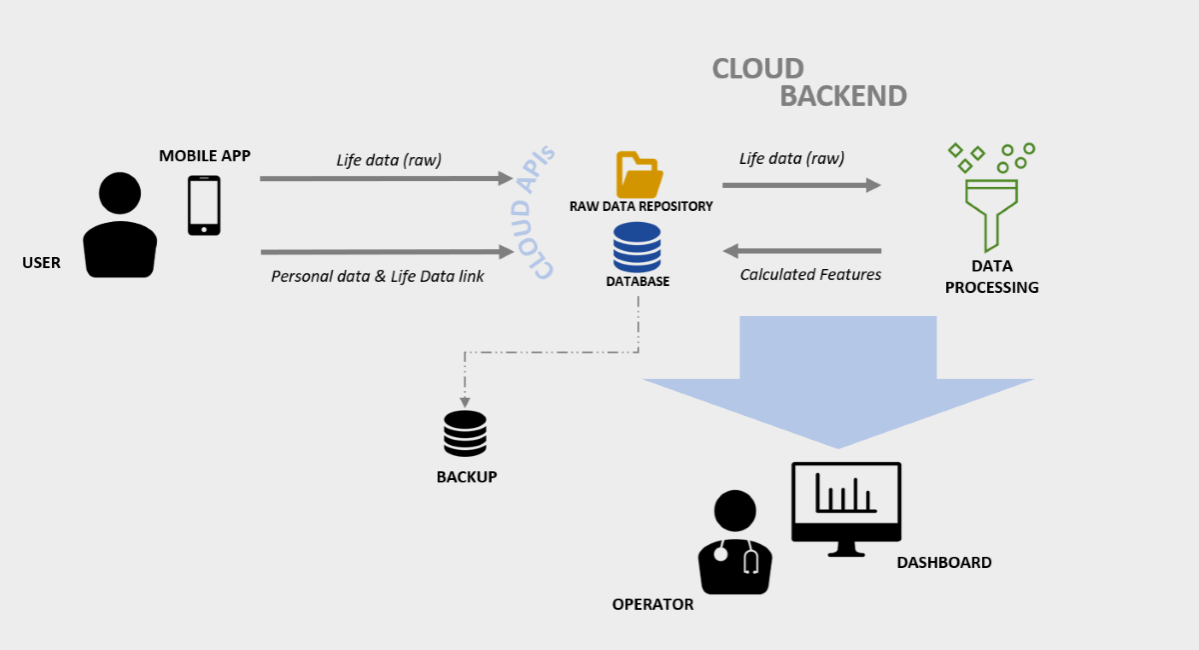
QuantifyMyPerson architecture consists of the user’s smartphone, a cloud backend for data storing and processing, and a web-based dashboard for data visualization.

The platform consists of a mobile app based on the Android OS, a cloud backend (backend as a service model), and a web-based dashboard (Figure 1). Data acquisition runs in the background during daily use of the smartphone by means of a passive motion sensor data acquisition approach. Through data processing algorithms, selected features are extracted to describe users’ life and behavior changes. The dashboard allows for management of the registry of users and visualization of the acquired data in graphical and numerical forms.

The monitoring app stores data locally on the smartphone and transmits them as Wi-Fi network connectivity becomes available. This strategy allows data collection in a variety of wireless connectivity scenarios with the confidence that intermittent network access does not affect the nature, quality, or quantity of the collected data.

**Figure 2:**
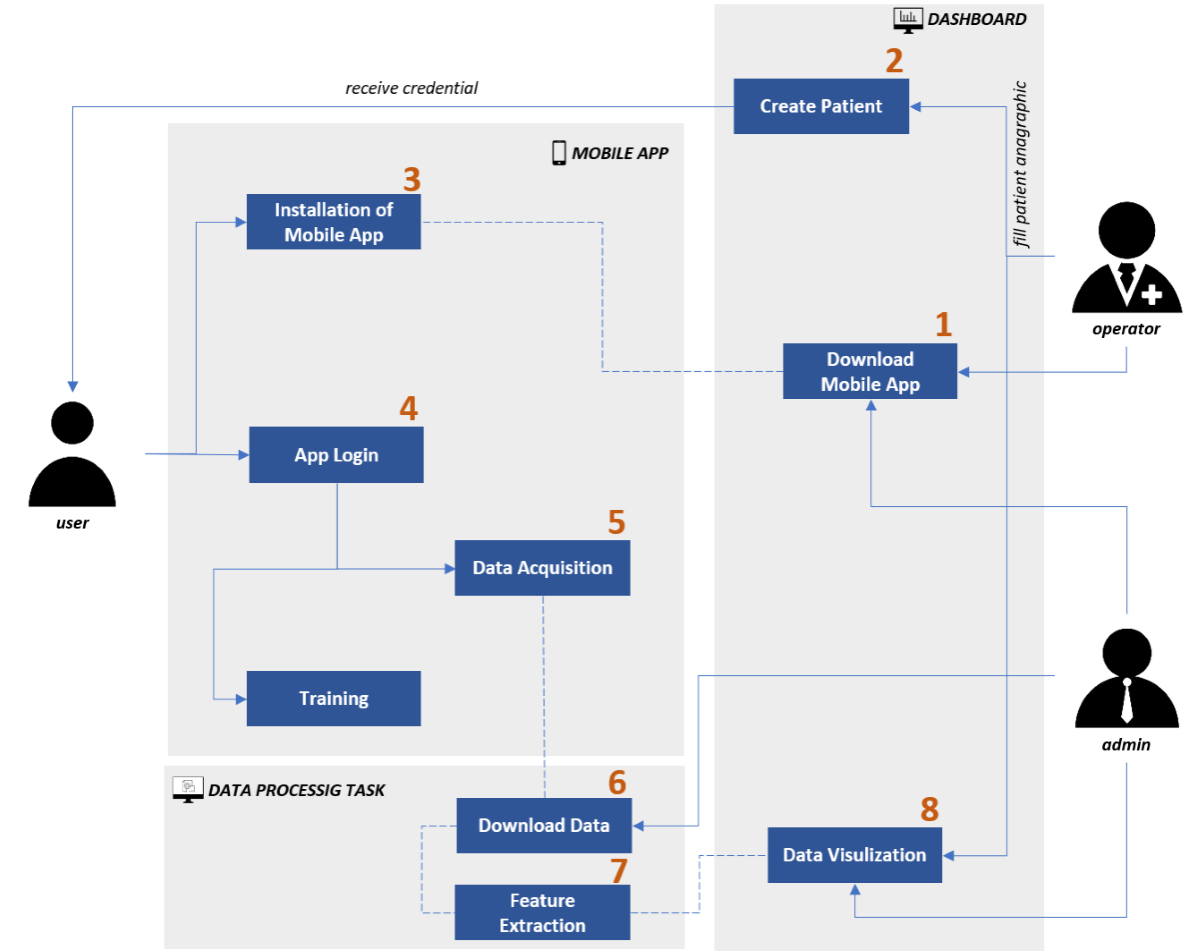
QuantifyMyPerson data flow from app installation to data visualization.

Acquired data are temporarily saved in a remote storage area and processed daily to extract descriptive features. The computed features are saved on an in-cloud database accessed through a web-based dashboard. The dashboard, as a management tool, makes available various means for the management of the patient database while acquired features may be displayed through different graph typologies on freely selectable time windows. The use flow is described in Figure 2.

The mobile app is the core element of the sensing platform. During the study, the app was available for Android platforms only as this operating system enables more flexibility in the management and access to the data registries and sensors. The mobile app uses the sensor made available by the Android framework to interact with the inertial measurement unit sensors embedded in the smartphone and with the social and communication registries made available by the operating system. The sensors to be used for collecting data and related sampling frequencies can be set through a parametric configuration table allowing a dynamic fine tuning of the acquisition parameters without the need for updating the app on users’ devices.

The following embedded sensors and registries are used as raw data sources by the QMP mobile app:

- Accelerometer sensor
- Gyroscope sensor
- Barometer sensor
- Magnetometer sensor
- GPS sensor
- Exchanged kBs per app registry
- Calls log registry
- Short message service log registry
- Screen brightness registry

The data acquired through the sensors are locally stored on the device as separated raw text files, one for each sensor. The raw files are then sent through an https encrypted communication protocol when a Wi-Fi connection is available in order to reduce the internal memory occupation and user data plan consumption. After the first log-in, the app runs in the background without any intervention from the user.

### Data Processing

The features extracted from the raw data are based on the main findings reported in the literature according to a previous review of ours [27]. Table 1 summarizes the features calculated from raw data.

**Table 1:** Behavioral features extracted by QuantifyMyPerson.

Feature		Description
**Calls**		
	mean_incoming	Average duration of incoming calls (seconds)
	mean_outgoing	Average duration of outgoing calls (seconds)
	tot_call_length	Total call duration (seconds)
	outgoing_call	Number of calls made
**Brightness**		
	mean_time_usage	Average duration of a session of use (from screen switch on to screen switch off)
	number_switch_on	Number of times screen is switched on
	b_*n* (24)	Seconds of phone’s use from hour *n–1* to *n* with hourly granularity over the whole 24 hours
**Apps**		
	tot_kb_social	Kilobytes consumed in social app (Facebook, Instagram, Twitter, LinkedIn)
	tot_kb_communication	Kilobytes consumed in communication app (WhatsApp, Messenger, Telegram, Skype, Hangouts)
	tot_kb_navigation	Kilobytes consumed in navigation app (Chrome, Firefox, proprietary browser, Google, YouTube, Tripadvisor)
	tot_kb	Total kilobytes consumed in a day
**GPS**		
	number_of_clusters	Number of places visited
	time_outside	Percentage of time spent outside the home
	location_variance	Variability in a participant’s location calculated as location_variance = *(σ^2^_long_ + σ^2^_lat_)*, where *σ^2^_long_* and *σ^2^_lat_* represent the variance of the longitude and latitude, respectively, of the GPS location coordinates
	Entropy	Measure of how uniformly a participant spends time at different locations. Let *p_i_* denote the percentage of time that a participant spends in location cluster *i*. The entropy of the participant is calculated as entropy = −(p_i_*log(p_i_))
	visited_clusters	Latitude and longitude coordinates of the visited places according to the distance from home
**Activity**		
	m_amp_*n* (24)	Average of the acceleration signal amplitude from hour *n*–1 to *n* with hourly granularity over the whole 24 hours
	s_a_*n* (24)	Seconds of high activity from hour *n*–1 to *n* with hourly granularity over the whole 24 hours
	s_r_*n* (24)	Seconds of low activity from hour *n*–1 to *n* with hourly granularity over the whole 24 hours
	percentage_activity	High activity/(high activity + low activity)

### Study Design

A sample of 12 healthy people was recruited for this initial feasibility study for a time span of 7 months. As the aim of this study is to assess how smartphone-based passive sensing platforms cope with heterogeneous and complex environment, any Android user was considered eligible irrespective of the smartphone model, connection availability, or digital literacy. The final goal was to highlight any possible criticality that could occur during normal use under free-living conditions.

The participants’ smartphones included 5 different smartphone brands and 11 different models running Android operating system versions from 4.4 to 7. The brand distribution shows a prevalence of Samsung and Huawei devices. The mean age of the selected participants was 39 (SD 5.4) years, the majority were male (8/12, 67%), and the average number of days of use was 62.

All participants were informed of the study aims and modalities when installing and running the app. Data handling was fully compliant with the General Data Protection Regulation. To ensure proper awareness about the acquired and stored data and aim of the study, an in-app communication approach was adopted consisting of an interactive wizard that describes the data sources used and the scope of the study. This approach ensures proper communication about data management and study aims through a clear description that can be understood by everyone regardless of the digital literacy of the enrolled subject.

Each participant’s identity was pseudoanonymized with a random user ID, keeping the ID map separated from all other acquired data so that data cannot be traced back to individuals. Participant data were uploaded on a secured server using encrypted SSL protocol to ensure they cannot be intercepted by third parties. When people left the study, their personal data were removed while the raw data acquired during the study and the calculated features remain anonymously stored for scientific research purposes.

## Results

### Data Acquisition System

The system was first assessed in terms of performance with the 2-fold objective of evaluating the impact of the mobile app on the daily use of the smartphone and quality of the acquired data. As reported in the study description section, the device brand distribution spanned the most relevant Android market players. Samsung devices showed a prevalence of 33% (4/12) within the considered sample followed by Huawei devices with 25% (3/12), Xiaomi (8%), Asus (17%), and Honor (17%). This population distribution is fairly aligned with the brand fragmentation reported in the Android Fragmentation report [28] and the most recent Statista’s global smartphone market share [29]. Thus, the sample under analysis mirrors the Android user population making the recruited participants a representative sample.

During the validation phase, no issues concerning normal smartphone functioning were reported, and the everyday use of the smartphone was not hindered by the background activity of the app. The app was installed on the user smartphone without impacting the running of already installed apps. No lags or limits in functionalities were reported during the study period.

**Figure 3:**
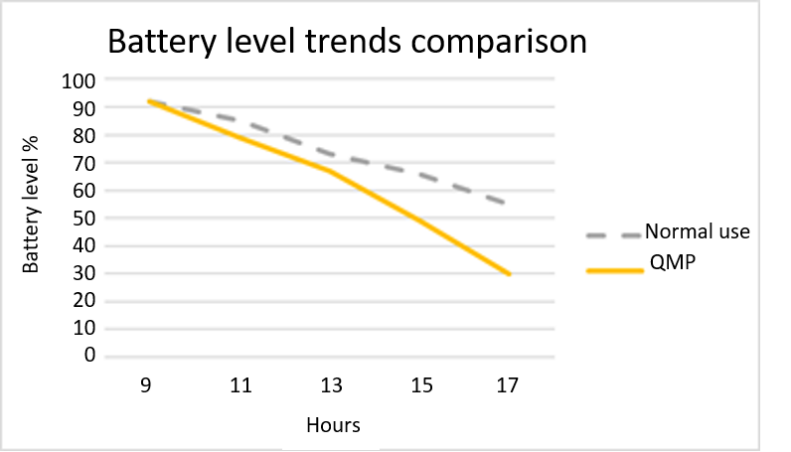
Comparison of the average daily battery consumption trend for the same smartphone with and without QuantifyMyPerson on board and running.

The average battery consumption trend of the smartphones, with and without the monitoring app on board, are compared in Figure 3. The battery drain analysis was made comparing performance within the same operational environments (running apps, operative system, connection type). The two trends are comparable with an acceptable increase in power consumption when the app is up and running. This trend confirms the known battery drain issues for smartphone-based passive sensing platforms, but the battery consumption can be well managed by tuning acquisition parameters such as sensors sample rate, data writing frequency, and data sending frequency.

Because of the implementation by some manufacturers of battery consumption management policies, some commercial devices do not allow continuous data acquisition from both the phone’s register and embedded sensors. This aspect could negatively influence reproducibility and scalability of smartphone-based monitoring systems especially within the Android ecosystem due to high level of fragmentation (brand, devices, and OS versions) if compared with the iOS systems [28].

**Figure 4:**
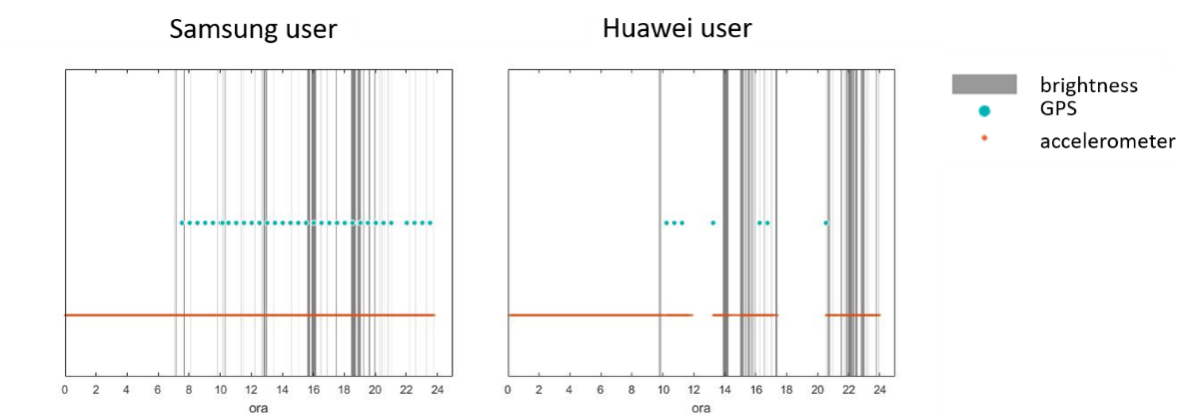
Comparison between the integrity of acquired data between a subgroup A user and a subgroup B user. Grey bars represent the timespan in which the brightness of the display has been detected, blue points identify GPS data collection, and the orange line comprises 5 Hz sampled embedded sensors data.

By analyzing the up time of each sensor within the selected population of users during the acquisition period, we identified two subgroups based on the behavior of their smartphone: subgroup A consisted of 7 users for whom the specific policies of the phone operating system do not impact on the continuity of data; subgroup B consisted of 5 users with smartphones whose proprietary operating system policies have a strong impact on the continuity of acquisition (Table 2 and Figure 4).

The most widely used battery consumption management policies switch the embedded sensors off when the phone is not used (ie, when the screen is off for some time) and when there are no changes in the GPS signal (when GPS is active). The phone is woken up again when one of the two situations changes.

This behavior makes clear that data from embedded sensors are lost mainly when the phone is still (ie, it is not used, and it is not moved). That means that most of the lost data from sensors might be not associated with periods of activity (assuming that users are carrying the phone on their person). So, the related loss of information should be not relevant, but more focused tests are still needed to validate this first outcome.

**Table 2:** Classification of user device within subgroup A and B according to the proprietary smartphone management policies.

Subgroup	Device brand and model
A	
	Asus Zenfone 4
	Samsung S4
	Samsung S6
	ASUS Zenfone 2 Laser
	Samsung S5
	Redmi 3S
B	
	Honor 8
	Huawei P10 Lite
	Honor 7
	Huawei P9 Lite
	Huawei P8 Lite

The quality and quantity of data are the main strengths of a continuous monitoring approach. Smartphone sensor issues, memory leaks, poor connection quality, and smartphone use in free-living condition have real impact on the quality of the collected data regardless the specific brand of the smartphone.

To assess these aspects, the data integrity percentage has been calculated using the following formula:



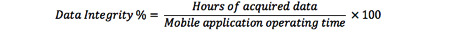



This measure is aimed at quantifying the percentage of data actually acquired while the app runs. The hours of acquired data are considered as the timespan during which the samples are acquired without interruption greater than 1 second. This measure provides an indirect computation of data samples lost during an acquisition session and allows us to spot gaps in the data.

Data integrity is a crucial parameter for the identification of the most appropriate data processing and feature extraction techniques. Datasets with a very low data integrity index should be not considered for frequency-based processing techniques or proper resampling techniques should be implemented.

This parameter might be used as a quality control parameter before mathematically or visually analyzing data. This approach should also be considered to ensure compliance with the medical device regulation (EU) 2017/745 on the risk of data misuse for clinical evidence extraction.

Within this study, the global data integrity percentage is 84% considering the entire sample of users, but a slightly different behavior was observed between the two subgroups of users described in the previous section. In particular, subgroup B is characterized by a lower value of data integrity percentage (60%) compared with subgroup A (96%). The analysis performed on the accelerometer, brightness, and GPS signals reveals that the lower data integrity percentage observed for the subgroup B of users is due to the previously described acquisition holes during which the smartphone kills the acquisition routine according to proprietary power-saving policies. At this time, no solutions have been found due to the proprietary policies implemented that are business confidential and differ between smartphone models and brands.

Data integrity has not been negatively impacted by poor or absent Wi-Fi connections and in some cases all the data were properly stored and sent even if the smartphone was not connected to Wi-Fi for a few days (up to 3). The data transfer protocol has proved to be efficient and capable of handling unreliable connections.

Thus, the strategy adopted for sending large raw data files based on file chunks periodically sent and attached to the master file stored on the remote repository provides a reliable data exchange protocol. This strategy allows decentralization of the computational power needed and thus reduces the impact on performance of the users’ smartphones. This approach is largely used for the management of data collection through a big data approach, and it is at the core of the edge computing paradigm that allows the implementation of sparse technological frameworks.

### Data Processing

Even if the focus of this study is not the clinical evaluation of the smartphone-based passive sensing platform, the collected data have been processed with the aim of extracting the features identified in the literature and evaluating their potentialities in identifying behavior trends and shifts for the analyzed users. The processing task was executed daily through an automatic routine.

The computed features were analyzed in order to investigate the information content and assess whether the typology and integrity of the available data could match with the data processing requirements for the analysis of trends and anomalies about human behavior. First, a principal component analysis was performed on the whole pool of features extracted with the aim of detecting the most descriptive set of features. The following features were identified as the most descriptive:

- GPS-related features such as movement index, location variance, and normalized entropy
- Hourly activity features—in particular, in the timespan that goes from 10 AM to 9 PM
- Hourly brightness features—in particular, in the timespan that goes from 11 AM to 10 PM

**Figure 5:**
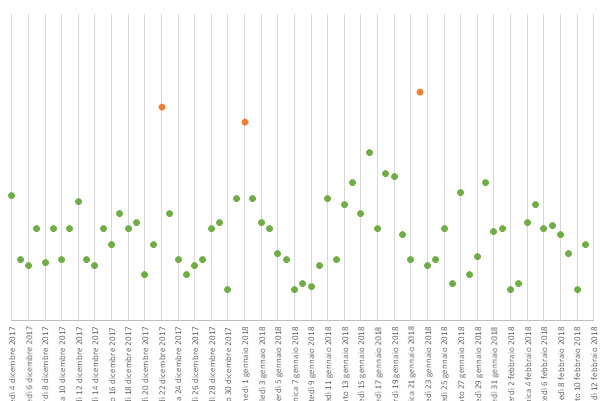
k-Nearest Neighbors Global Anomaly Score graph for a selected user. Points represent the calculated anomaly score for each day with the red points representing days with the highest values of anomaly.

Thus, the selected features were used to extract information about the variance between each day and detect anomalous days. This approach starts from the evidence reported by Berrouiguet [30] on the analysis of GPS-based features and validates its feasibility with a larger set of features than the ones investigated here. The set of features was analyzed using a k-Nearest Neighbor Global Anomaly Score in order to detect the days that differ from others within the period of use of QMP (Figure 5). This analysis showed a repeatable pattern for each user along the period of acquisition discriminating between the nonworking days and the working days. Public holidays instead were detected as the most relevant outliers showing the ability of the system to easily detect the days that differ most because of nonstandard behavior of the user.

People use their smartphones for different purposes and through different interaction modalities regardless their of demographic data, which has been demonstrated to not have relevant impacts on the smartphone-related behavior [31] Smartphones are strongly integrated in every aspect of people’s lives acting as a reliable behavioral mirror [32] enabling the longitudinal monitoring and indirect assessment of cognitive and physical status. Further analyses are ongoing in order to better assess the potentialities of identifying clinically relevant and behavior-related trends through a wider clinical trial. The first data analysis shows promising results for users belonging to both subgroup A and B. However, further investigation is still needed in order to find the best data normalization method that takes advantage of the data integrity parameters for each of the identified subgroups.

## Discussion

### Principal Findings

In this paper, we presented the results of a validation test aimed to assess the reliability of smartphone-based passive sensor systems and the related integrity of the acquired data. Also, a preliminary assessment of the informative content of these data and their correlation to real user behavior has been presented with the aim of associating the data integrity with the capability to extract valuable insights from data.

All participants used the system consistently and actively for the period of the study without any kind of technological constraint. The proposed approach proved to be able to manage the flow of data correctly without a consistent loss of information and provide a daily update of the calculated features. The method used to acquire data from the embedded sensors through a mobile app was able to work with the heterogenous and complex technical environment ensuring a good level of reliability and maintaining a good level of performance of the users’ smartphones without impacting the already installed apps. The system also registered a high level of acceptability due to the good level of integration in the normal use of the smartphone in conjunction with an adequate level of transparency and ubiquitousness that ensured reliable and meaningful results. Two participants (17%) asked to interrupt the study after 1 month due to battery drain effects, but the users continued the study when the battery drain effect was mitigated by activating the GPS sensor only for limited time spans within the day.

The current smartphone evolution is highly focused on the optimization of the battery consumption for the most energy-consuming sensors such as GPS and Bluetooth as many of the most common apps require their continuous running (eg, COVID-19 tracking apps). Thus, the impact on battery drain is also expected to be reduced for passive sensor platforms such as the one used in this study. Besides, the use of monitoring tools in the frame of a structured digital health approach will further justify the power consumption side effect thanks to demonstrated care benefits.

In this study, data acquisition, performed by means of the users’ own smartphones without any limitations or technological eligibility criteria, reached a remarkable integrity of the globally acquired data—14,970 hours of collected sample out of 17,845 hours of acquisition (84%)—that surpasses the performances presented in previous publications [33], proving the potentialities of passive sensing platforms. However, different behaviors observed for subgroup A and B have some impacts on the data integrity ratio with 96% and 60%, respectively, when kept separately. Brand-related operating system policies still have the most important impact on data integrity due to the observed fragmentation of the Android services.

The availability of different sensor data allows us to describe each subject in terms of physical activity (accelerometer data), social interaction (calls, communications, and social network data), and georeferenced data. This approach provides an overall description of each user that can be used to continuously monitor both the psychological and physical status, strengthening the added value of this type of system which can provide a comprehensive description of quality of life and well-being. The wide range of data made available by monitoring platforms can also be considered the necessary starting point for data fusion approaches [34].

Preliminary analysis of the obtained results shows that the data fusion between different sensors provides a valuable key to interpret personal behavior. In particular, the demonstrated capability of identifying anomalous days is strongly dependent on the variability in content of the acquired data and can represent a strong starting point for different clinical applications. Furthermore, the habits about smartphone use itself could be used as a valid behavioral descriptors. For example, intrapersonal changes in frequency and duration of smartphone daily usage or the inactivity period of the smartphone due to the fact the user has not carried the smartphone can be used as indirect behavioral descriptors. Thus, the fusion between data acquired from different contexts gives a comprehensive description considering all the aspects that can be impacted by changing physical or psychological condition. Also, the smartphone use parameters (eg, screen time, battery use) can be used to normalize the calculated features, reducing the bias due to different smartphone use that can impact the reliability of the collected data.

### Limitations

This study has been conducted on a limited number of subjects with a focus on Android devices. A wider study should be conducted including a wider pool of devices and subjects. Furthermore, assessment of the approach on acute and chronic patients is required to ensure generalizability in the clinical application domain.

The use of mobile apps for health monitoring is still in an early phase. To foster their acceptance at a wider level, making the collected information routinely useful for the health care system, clinical validations are necessary to select the best parameters to investigate each pathological condition. However, analysis of this aspect was outside the scope of this study, which focused on assessment of perceived technical limitations to daily use.

### Conclusion

In this paper, we present a study that contributes as an additional step to broad distribution of smartphone-based monitoring platforms. We described the technical approach used to implement a smartphone-based passive platform, its characteristics, and the potentialities of this type of solution to provide insights to patients and clinicians. The quality of the acquired data and performance of the system are quite dependent on the proprietary policies implemented by each smartphone brand even if the acquisition through smartphone-embedded sensors as presented in this article is able to provide a good level of accuracy within an heterogenous pool of devices. The preliminary analysis performed on the raw data collected provides initial encouraging results that must be better validated through well-structured clinical trials with the aim of substantiating the clinical evidence of monitoring systems and their capability of extracting indexes that could be used as reliable descriptors and predictors of the disease path.

In the future, we will continue this work deepening the technical validation of this type of platform to assess performance and quality of the collected data on a wider study cohort including the most recent Android updates and newest smartphone brands. Furthermore, the research will focus on the assessment of the data fusion potentialities for the extraction of valuable clinical insights according to the characteristics of the collected data. Additionally, as the performance of this type of monitoring system is quite depending on the policies of each smartphone producer, a wider discussion could address guidelines that could match with the needs of mHealth in the near future.

Discussion about the repeatability and reliability of smartphone-based passive sensing platforms should also drive the debate about software as medical device and its applicability in the current regulatory framework. This is still an open issue [35] whose resolution will be necessary to drive the successful use of monitoring systems as scalable and reliable supports for the clinical practice. Also, ethical and security aspects will be investigated to make the system as secure as possible by design. Thus, a quantified technical characterization of the system in terms of reproducibility and robustness of the provided measurements will be necessary, and the proposed article could be considered a good methodologic starting point.

## Abbreviations

CIMON: Crew Interactive MObile CompanioN
mHealth: mobile health
QMP: QuantifyMyPerson
